# Analysis of DNA methylation acquisition at the imprinted *Dlk1* locus reveals asymmetry at CpG dyads

**DOI:** 10.1186/1756-8935-7-9

**Published:** 2014-05-29

**Authors:** Alyssa Gagne, Abigail Hochman, Mahvish Qureshi, Celia Tong, Jessica Arbon, Kayla McDaniel, Tamara L Davis

**Affiliations:** 1 Department of Biology, Bryn Mawr College, 101 N. Merion Avenue, Bryn Mawr, PA 19010-2899, USA

**Keywords:** Genomic imprinting, DNA methylation, *Dlk1*, Secondary DMR, Epigenetics

## Abstract

**Background:**

Differential distribution of DNA methylation on the parental alleles of imprinted genes distinguishes the alleles from each other and dictates their parent of origin-specific expression patterns. While differential DNA methylation at primary imprinting control regions is inherited via the gametes, additional allele-specific DNA methylation is acquired at secondary sites during embryonic development and plays a role in the maintenance of genomic imprinting. The precise mechanisms by which this somatic DNA methylation is established at secondary sites are not well defined and may vary as methylation acquisition at these sites occurs at different times for genes in different imprinting clusters.

**Results:**

In this study, we show that there is also variability in the timing of somatic DNA methylation acquisition at multiple sites within a single imprinting cluster. Paternal allele-specific DNA methylation is initially acquired at similar stages of post-implantation development at the linked *Dlk1* and *Gtl2* differentially methylated regions (DMRs). In contrast, unlike the *Gtl2*-DMR, the maternal *Dlk1*-DMR acquires DNA methylation in adult tissues.

**Conclusions:**

These data suggest that the acquisition of DNA methylation across the *Dlk1/Gtl2* imprinting cluster is variable. We further found that the *Dlk1* differentially methylated region displays low DNA methylation fidelity, as evidenced by the presence of hemimethylation at approximately one-third of the methylated CpG dyads. We hypothesize that the maintenance of DNA methylation may be less efficient at secondary differentially methylated sites than at primary imprinting control regions.

## Background

Genomic imprinting in mammals results in the monoallelic expression of approximately 150 genes [1,2]. The majority of these imprinted genes are found in clusters distributed throughout the mammalian genome, with each cluster containing two or more imprinted genes as well as an imprinting control region (ICR) [3]. One common feature of the CpG-rich ICRs is the presence of a gametic, or primary, differentially methylated region (DMR) which generally functions both to identify parental origin and to regulate expression of the imprinted genes within the cluster, either directly or indirectly [3]. Establishment of parent of origin-specific DNA methylation at the ICR occurs during gametogenesis and the zygote either inherits a methylated allele from its mother or from its father at fertilization. Differential methylation at the ICR is then maintained throughout development such that the parental alleles can be distinguished from each other and the expression of their adjacent imprinted genes regulated appropriately.

In addition to the differential methylation present at the ICR, some imprinted loci also acquire distinct secondary regions of differential methylation during post-implantation development [4-6]. It has been proposed that the establishment of differential DNA methylation at secondary DMRs could serve as a mechanism for maintaining imprinted expression at developmental times when the primary imprinting control region is no longer functioning [6,7]. Support for this hypothesis comes from a recent study of DNA methylation and expression at the imprinted *Gpr1-Zdbf2* locus at which the maternally methylated *Gpr1* DMR functions as the gametic imprinting mark responsible for establishing paternal allele-specific expression while paternal allele-specific DNA methylation at the secondary *Zdbf2* DMR is established after the onset of imprinted *Zdbf2* expression [8]. Paternal allele-specific expression of *Zdbf2* is maintained after DNA methylation at the *Gpr1* DMR becomes biallelic, suggesting that the paternally methylated secondary *Zdbf2* DMR functions to maintain monoallelic expression at this locus. Furthermore, biallelic methylation at the *Zdbf2* DMR in offspring derived from *Dnmt3L*^*mat-/-*^ mothers correlated with biallelic expression of *Zdbf2*. While the exact mechanism responsible for the parental allele-specific acquisition of DNA methylation at secondary DMRs has not yet been determined, it is clear that there is a relationship between the epigenetic states at primary and secondary DMRs [9,10].

The majority of secondary DMRs found at imprinted genes are methylated on the paternally-inherited allele, suggesting that there may be a common mechanism responsible for establishing secondary imprinting marks. At the same time, it is clear that not all secondary DMRs are acquired at the same developmental stage. Paternal allele-specific DNA methylation is established at *Gtl2* prior to 6.5 days post coitum (d.p.c.), at *Cdkn1c* between 7.5 and 9.5 d.p.c. and at *Igf2r* region 1 during late embryogenesis [7,11-13]. *Gtl2*, *Cdkn1c*, and *Igf2r* are located on mouse chromosomes 12, 7, and 17, respectively. DNA methylation at secondary DMRs has generally been shown to affect the expression of a single adjacent imprinted gene, rather than the expression of the entire imprinting cluster [6,7]. Therefore, it is possible that the same molecular machinery is used to establish DNA methylation at these sites and that the difference in temporal acquisition reflects the time at which it becomes critical to maintain monoallelic expression for each imprinted gene.

The *Dlk1-Dio3* cluster of imprinted genes spans 1 Mb on mouse chromosome 12 and contains three paternally expressed protein-coding genes (*Dlk1*, *Rtl1*, and *Dio3*), multiple maternally expressed untranslated RNAs (including *Gtl2*), and at least three DMRs that are methylated on the paternal allele [14-18]. The IG-DMR, located between *Dlk1* and *Gtl2*, functions as the ICR on the unmethylated maternally inherited allele [19]. Secondary DMRs have been identified at the promoter of *Gtl2* and in exon 5 of *Dlk1*[5]. Evidence suggests that the *Gtl2*-DMR has a functional role; studies of the mouse *Gtl2*-DMR and its human homolog, *MEG3*-DMR, indicate that methylation of this region directly influences expression *in cis*[10,20,21]. Although the functional role of differential methylation at *Dlk1* has not been determined, both the *Gtl2*- and *Dlk1*-DMRs become methylated on the paternal allele following fertilization, and the *Gtl2*-DMR has been shown to acquire paternal allele-specific methylation during early post-implantation development, between embryonic days 3.5 and 6.5 [5,11]. Since these two DMRs are located within the same imprinting cluster, we hypothesized that the acquisition of paternal allele-specific DNA methylation at these secondary DMRs would be coordinately controlled. We tested this hypothesis by examining the methylation status of the *Dlk1*-DMR throughout development. We found that the *Dlk1*-DMR acquires paternal allele-specific methylation during embryogenesis and that the methylation pattern remains dynamic in late embryonic development and into adulthood. Furthermore, our analysis of DNA methylation on the complementary strands of the *Dlk1*-DMR illustrates the unexpectedly fluid nature of DNA methylation at this locus.

## Results

### The *Dlk1*-DMR acquires paternal allele-specific DNA methylation during post-fertilization development

Previous research illustrated that somatic mouse tissues exhibit paternal allele-specific DNA methylation at the *Dlk1*-DMR that is acquired after fertilization [5,14,15]. To elucidate the temporal acquisition of paternal allele-specific DNA methylation at the *Dlk1*-DMR following fertilization, we assessed the DNA methylation status on both the paternal and maternal *Dlk1* alleles at various stages of mouse development.

**Figure 1:**
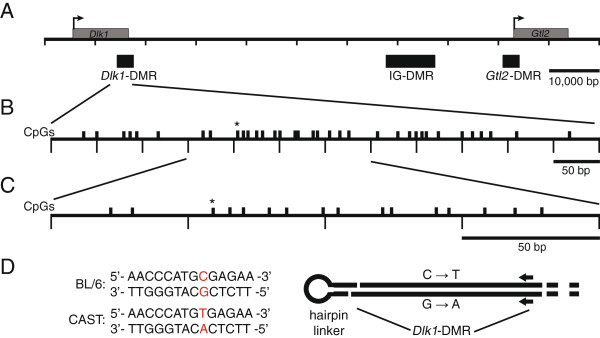
**Schematic of *Dlk1-Gtl2 *imprinting cluster and regions analyzed. (A)***Dlk1-Gtl2* imprinting cluster, including transcriptional start sites (arrows), transcription units (gray boxes) and differentially methylated regions (black boxes). **(B, C)** The 600 bp (B) and 220 bp (C) regions of the *Dlk1*-DMR analyzed by bisulfite mutagenesis and DNA sequencing in this study, corresponding to positions 109,459,577-109,460,173 and 109,459,680-109,459,900, NC_000078.6, respectively. The C/T polymorphism (*) between C57BL/6 J and *Mus musculus castaneus* is located at 109,459,746. **(D)** Sequence flanking the C/T polymorphism (red text) and schematic representing ligation of the hairpin linker to *Bgl*I-digested genomic DNA. The hairpin linker was designed to anneal to itself, forming a hairpin structure, and to the 3′ overhang generated following *Bgl*I digestion. Ligation of the hairpin linked to *Bgl*I-digested DNA results in the covalent attachment of the complimentary strands of DNA. Primers (block arrows) were designed to anneal to the bisulfite-mutagenized genomic DNA in order to amplify the region of interest.

All of our experiments were conducted using F_1_ hybrid tissues collected from crosses between C57BL/6 (B6) and a specially derived strain containing *Mus musculus castaneus*-derived sequences from chromosome 12 on an otherwise C57BL/6 genetic background (CAST12) [11]. We identified a single nucleotide polymorphism between the B6 and CAST12 strains in a 386 bp CpG island located at the 5′ end of *Dlk1* exon 5 (http://www.ebi.ac.uk/Tools/emboss/cpgplot/index.html) [11]. The identified SNP was a C-to-T transition at base pair position 109,459,746 (GenBank: NC_000078.6), preventing us from definitively assigning parental origin following bisulfite mutagenesis and sequencing of the top strand of DNA, since unmethylated cytosines would ultimately be replaced by thymines. Therefore, we modified our approach by covalently attaching the top and bottom strands via a hairpin linker, which allowed us to identify parental origin based on the G-to-A transition on the bottom strand (Figure 1D; see Methods). This approach had the additional advantage of yielding DNA methylation data for complementary CpG dinucleotides, allowing us to determine the level of homo- *versus* hemimethylation within this region. We used this approach to analyze the methylation status of 16 of the 29 CpGs located within the *Dlk1* CpG island (Figure 1C).

**Figure 2:**
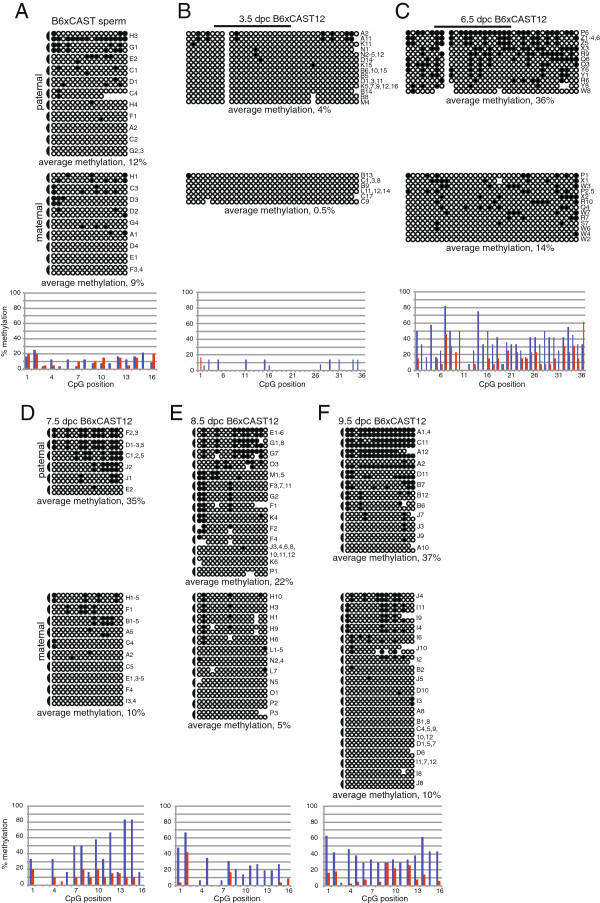
**Paternal allele-specific DNA methylation is acquired during post-implantation development. (A)** Bisulfite mutagenesis and sequencing of DNA from B6 x CAST F_1_ hybrid spermatozoa. **(B-F)** Bisulfite mutagenesis and sequencing of DNA from B6 x CAST12 F_1_ hybrid 3.5 to 9.5 d.p.c. embryos. A hairpin linker approach was used to analyze the DNA methylation status of 32 potentially methylated CpG dinucleotides in sperm and 7.5 to 9.5 d.p.c. embryos. Individual circles represent one of the 32 potentially methylated CpG dinucleotides, and each paired row of circles represents the complimentary strands of an individual subclone; semi-circles to the left connect the complimentary strands. A traditional bisulfite mutagenesis approach was utilized to analyze 36 CpG sites located on the bottom, antisense strand in 3.5 d.p.c blastocysts and 6.5 d.p.c. embryos; the analyzed region includes all 29 sites contained within the *Dlk1* CpG island and all 16 sites analyzed using the hairpin linker (black bar). The gap in the paternal strands represents the polymorphic site, which is not a CpG dinucleotide in *Mus musculus castaneus*-derived DNA. Filled circles represent methylated cytosines, open circles represent unmethylated cytosines, absent circles represent ambiguous data. Labels to the right identify the PCR subclone analyzed; letters represent independent amplification reactions, while numbers represent individual subclones. The level of methylation (y axis) on the paternal (blue) and maternal (red) strands was quantified by dividing the number of methylated residues at each CpG site by the total number of sites analyzed at that position.

We confirmed that adult sperm DNA contains very low levels of DNA methylation at the *Dlk1*-DMR (Figure 2A). Therefore, any paternal allele-specific methylation observed in somatic tissues must be acquired during post-fertilization development. To determine when DNA methylation is acquired at the *Dlk1*-DMR, we analyzed the methylation status at the *Dlk1*-DMR during early embryonic development. We were unable to scale down the hairpin linker approach for use with the limited amount of material collected from 3.5 d.p.c. blastocysts and 6.5 d.p.c. embryos. Therefore, for these developmental stages we utilized a traditional bisulfite mutagenesis approach to analyze the DNA methylation status at 36 CpG sites, including all 29 sites contained within the *Dlk1* CpG island and all 16 sites analyzed using the hairpin linker employed for analysis of DNA derived from older embryonic, neonatal, and adult tissue (Figure 1). We observed an absence of DNA methylation on both the paternal and maternal alleles in 3.5 d.p.c. blastocysts, indicating that the paternal *Dlk1* allele does not acquire methylation during pre-implantation development (Figure 2B). By 6.5 d.p.c., the paternal *Dlk1* allele has acquired DNA methylation (Figure 2C). We assessed the significance of these results using a Mann–Whitney U test and found that there was a statistically significant difference in the median level of DNA methylation on the paternal alleles of 3.5 vs. 6.5 d.p.c. embryos (*P* <0.0001). Although the level of DNA methylation on maternal alleles also increases significantly between 3.5 and 6.5 d.p.c. (*P* = 0.0023), the level of DNA methylation at the paternal *Dlk1*-DMR in 6.5 d.p.c. embryos is significantly higher than the level of methylation on maternal alleles (*P* = 0.0025), illustrating that differential DNA methylation has been established at the *Dlk1*-DMR by 6.5 d.p.c. All of the raw data used to conduct the Mann–Whitney U tests can be found in Additional file 1.

We next assessed DNA methylation in 7.5 to 9.5 d.p.c. embryos. While the average level of DNA methylation is somewhat variable in 6.5 to 9.5 d.p.c. embryos (Figure 2C-F), neither the variation observed between the paternal alleles at different developmental stages nor between the maternal alleles at different developmental stages was significant, although the paternal and maternal alleles remain different from each other. Average levels of DNA methylation for each of the parental alleles at all developmental stages analyzed are presented in Table 1; this information, along with medians and IQ ranges can be found in Additional file 2. These results demonstrate that the paternal allele-specific DNA methylation that is established at the *Dlk1*-DMR prior to 6.5 d.p.c. is maintained during early embryonic development. The timing of DNA methylation acquisition at the *Dlk1*-DMR is similar to that which we and others have observed at the paternal *Gtl2-*DMR [11,12], suggesting that the acquisition of DNA methylation across the *Dlk1/Gtl2* locus may be coordinately controlled.

**Table 1:** **Average levels of DNA methylation on the paternal and maternal *Dlk1*-DMR alleles during development**

**Genomic DNA sample**	**% methylation, paternal alleles**	**% methylation, maternal alleles**
B6xCAST adult sperm	12%	9%
3.5 d.p.c. B6xCAST12 embryo	4%	0.5%
6.5 d.p.c. B6xCAST12 embryo	36%	14%
7.5 d.p.c. B6xCAST12 embryo	35%	10%
8.5 d.p.c. B6xCAST12 embryo	22%	5%
9.5 d.p.c. B6xCAST12 embryo	37%	10%
14.5 d.p.c. B6xCAST12 embryo	27%	5%
14.5 d.p.c. CAST12xB6 embryo	21%	3%
17.5 d.p.c. CAST12xB6 liver	74%	18%
5 d.p.p. B6xCAST12 liver	46%	11%
5 d.p.p. CAST12xB6 liver	62%	24%
6 d.p.p. B6xCAST12 lung	45%	8%
5 d.p.p. CAST12xB6 lung	44%	14%
Adult B6xCAST12 liver	74%	57%
Adult CAST12xB6 liver	58%	41%
Adult B6xCAST12 lung	18%	6%
Adult CAST12xB6 lung	53%	32%

Medians and IQ ranges for each of the average levels of DNA methylation are presented in Additional file 2. In addition, a Kruskal-Wallis test was used to assess overall variation among paternal alleles and among maternal alleles. Significant variation was observed among both the paternal alleles (*P* = 8.335e-13) and the maternal alleles (*P* = 1.844e-12).

### DNA methylation patterns at the *Dlk1*-DMR are dynamic during development

**Figure 3:**
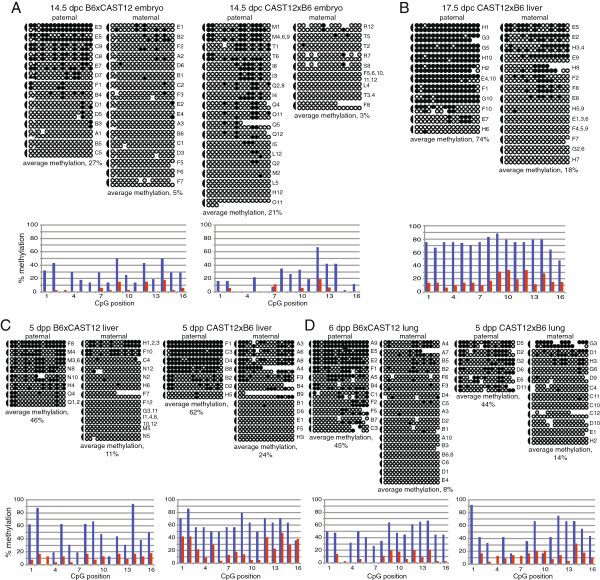
**Paternal allele-specific DNA methylation persists during embryonic and neonatal development.** Bisulfite mutagenesis and sequencing of DNA derived from B6 x CAST12 and CAST12 x B6 14.5 d.p.c. F_1_ hybrid embryos **(A)**, CAST12 x B6 17.5 d.p.c. F_1_ hybrid liver **(B)** and B6 x CAST12 and CAST12 x B6 5 to 6 day post partum F_1_ hybrid liver and lung tissue **(C, D)**. Details as described in Figure 2.

We next examined the DNA methylation status at the *Dlk1*-DMR in mid- and late-gestation embryos to investigate the progression of DNA methylation acquisition during later developmental stages. The level of methylation we observed on the paternal alleles of 14.5 d.p.c. embryos was similar to the earlier embryos (Figure 3A; Table 1), and statistically significant differences were not observed between 6.5 and 9.5 d.p.c. embryos *versus* 14.5 d.p.c. embryos. In contrast, 75% of the CpGs were methylated on paternal alleles derived from 17.5 d.p.c. liver (Figure 3B), and the median level of DNA methylation at this stage was significantly higher when compared to 6.5, 7.5, 8.5, 9.5, and 14.5 d.p.c. embryos (*P* = 0.0191, 0.0435, 0.0018, 0.0309, and 0.0005, respectively). In contrast, no statistically significant differences were detected when DNA methylation levels on the maternal alleles of 17.5 d.p.c. liver were compared to maternal alleles derived from earlier embryos. These data indicate that the DNA methylation status of the paternal *Dlk1*-DMR continues to be labile into late embryogenesis.

It had previously been reported that the extent of DNA methylation on the maternal and paternal alleles of *Dlk1* varied in different tissues [5]. We therefore examined the methylation status at the *Dlk1*-DMR in stage-matched neonatal liver and lung tissues derived from reciprocal crosses between B6 and CAST12 mice. We chose liver and lung as representative tissues for this analysis as these tissues exhibit low and high levels of *Dlk1* expression during perinatal development, respectively [5]. We found that the paternally inherited allele had a significantly higher level of DNA methylation than the maternally inherited allele in both B6xCAST12 and CAST12xB6 tissues (*P* = 0.001, liver; *P* <0.0001, lung; Figure 3C, D), consistent with previously obtained data derived from DNA methylation analyses of 18.5 d.p.c. uniparental disomic (UPD) 12 liver and lung tissues [5]. In addition, the median levels of DNA methylation on paternal alleles derived from neonatal liver and lung were significantly higher than the median levels in 14.5 d.p.c. embryos (*P* = 0.0016 and 0.004, respectively), indicating that the DNA methylation level continues to increase on the paternal allele during development. However, we did not detect statistically significant differences in the DNA methylation patterns of neonatal liver *versus* lung, demonstrating that the methylation status of *Dlk1* in these tissues is not different at this developmental stage.

**Figure 4:**
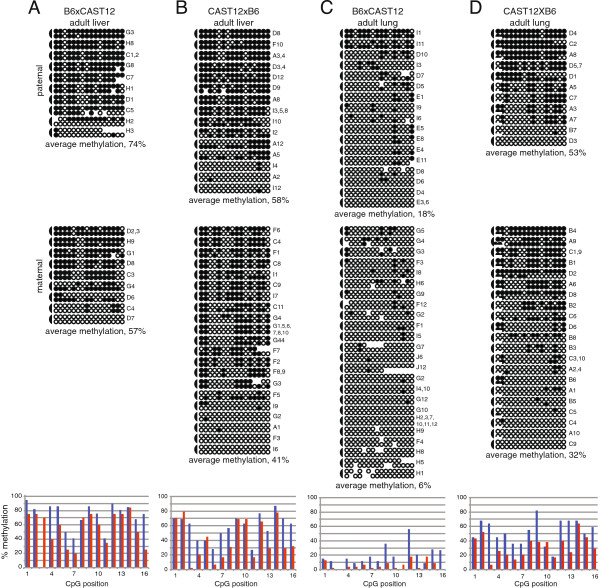
**DNA methylation is acquired on the maternal *Dlk1*-DMR in adult tissues.** Bisulfite mutagenesis and sequencing of DNA from B6 x CAST12 adult liver **(A)**, CAST12 x B6 adult liver **(B)**, B6 x CAST12 adult lung **(C)**, and CAST12 x B6 adult lung **(D)**. Details as described in Figure 2.

Surprisingly, when we analyzed DNA derived from adult B6xCAST12 liver, we found high levels of methylation on both the paternal (74%) and maternal alleles (57%) (Figure 4A). We hypothesized that the maternal *Dlk1* allele may acquire methylation at a later stage of development or that the hypermethylation we observed on the maternal allele was an adult tissue-specific pattern. We therefore examined the methylation pattern in DNA derived from adult CAST12xB6 liver as well as adult B6xCAST12 and CAST12xB6 lung. We observed relatively high levels of DNA methylation at the *Dlk1*-DMR on both parental alleles in adult CAST12xB6 liver, consistent with the DNA methylation profile we observed in adult B6xCAST12 liver (Figure 4A, B), supporting the hypothesis that acquisition of DNA methylation on the maternal *Dlk1* allele occurs during a later stage of development. While the CAST12xB6 adult lung tissue also displayed high levels of DNA methylation on both parental alleles, both the maternal and the paternal alleles of *Dlk1* were hypomethylated in adult B6xCAST12 lung tissue (Figure 4C, D). We did not anticipate the significant difference in DNA methylation status that we observed in the B6xCAST12 *versus* CAST12xB6 lung tissue, as none of the other data we obtained from stage-matched reciprocal crosses (14.5 d.p.c., 5 to 6 days post partum, and adult liver) displayed this variation. However, it is possible that this experiment uncovered a sensitivity to genetic background at the *Dlk1* locus. Further experiments are needed to determine the extent of DNA methylation variation in adult tissues. Regardless, from these results, we conclude that the DNA methylation status of the *Dlk1*-DMR continues to change during later stages of mouse development.

Our statistical analyses indicate that the low level of DNA methylation observed on the maternal *Dlk1*-DMR of 6.5 d.p.c. embryos does not change significantly during post-implantation and perinatal development, although it acquired significantly higher levels of DNA methylation in some of the adult tissues analyzed. In contrast, the median level of DNA methylation on the paternal *Dlk1*-DMR is significantly different in early and mid-gestation embryos when compared either to late embryos or to adult liver, illustrating that the paternal *Dlk1*-DMR becomes incrementally more methylated over time. Therefore, although the onset of paternal allele-specific DNA methylation acquisition at the *Dlk1*- and *Gtl2*-DMRs occurs at a similar time during development, the DNA methylation pattern at the *Dlk1*-DMR is more labile (Table 1).

### Placental tissue displays biallelic methylation at the *Dlk1*-DMR

**Figure 5:**
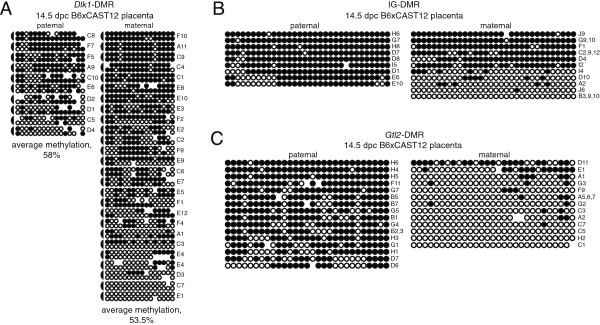
**DNA methylation of the paternally-inherited *Dlk1*-DMR is higher in the 14.5 d.p.c. placenta than in the 14.5 d.p.c. embryo, and is predominantly on the paternal allele at the IG- and *Gtl2*-DMRs in placenta.** Bisulfite mutagenesis and sequencing of DNA derived from B6 x CAST12 14.5 d.p.c. F_1_ hybrid placenta; details as described in Figure 2. **(A)** Analysis of DNA methylation at the *Dlk1*-DMR. For **(B)** and **(C)**, the regions analyzed in this study correspond to IG-DMR region 1 and *Gtl2*-DMR region 5 analyzed by Sato *et al*. [12]. **(B)** Each circle represents one of 32 potentially methylated CpG dinucleotides at the IG-DMR, the first one located at position 110,766,345 (NC_000078.5). **(C)** Each circle represents one of 29 potentially methylated CpG dinucleotides at the *Gtl2*-DMR, the first one located at position 110,779,349 (NC_000078.5).

To complete our analysis of the developmental dynamics of DNA methylation at *Dlk1*, we investigated the methylation status in 14.5 d.p.c. B6xCAST12 placenta. Fifty-eight percent of the CpGs were methylated on the paternal alleles and 53.5% were methylated on the maternal alleles, suggesting that both parental alleles are partially methylated in mouse placenta (Figure 5A). These data are consistent with those previously obtained using a methylation-sensitive southern blot to assess DNA methylation levels on the parental alleles of the *Dlk1*-DMR in 16.5 d.p.c. placentae [22]. While the median level of DNA methylation on the parental alleles in 14.5 d.p.c. placenta is significantly different from the level observed in the corresponding 14.5 d.p.c. embryo (27% and 5% for the paternal and maternal alleles, respectively; *P* <0.0005), previous research has shown that the *Gtl2*-DMR is methylated on both parental alleles in 6.5, 7.5, and 16.5 d.p.c. extraembryonic tissues, and it has been suggested that both the regulation of the non-coding RNAs and the maintenance of DNA methylation in the *Dlk1-Dio3* imprinting cluster differs in embryonic *versus* extraembryonic tissue [12,15,22].

Our analysis of methylation at the *Dlk1*-DMR was complicated by the fact that we did not separate the maternal component of the placenta from the embryonic component. Therefore, while paternal CAST12 alleles must be derived from the embryonic component of the placenta, B6 alleles could derive from the maternal allele in the embryonic component of the placenta or from either of the parental alleles in the maternal component. To assess the relative proportion of embryonic *versus* maternally-derived B6 DNA in our placental samples, we analyzed the methylation status at the IG-DMR, which has been shown to remain differentially methylated in extraembryonic tissue and placenta [12,22]. As expected, we detected hypermethylation on the nine paternal IG-DMR alleles we analyzed. In contrast, six of the 11 maternal alleles analyzed were hypermethylated, suggesting that these hypermethylated alleles were derived from the maternal component of the placenta and that the level of DNA methylation we observed on the maternal alleles overestimates the true extent of methylation present on the maternal *Dlk1* alleles in the placenta (Figure 5B). Interestingly, we observed differential methylation on the parental *Gtl2*-DMR alleles in the same 14.5 d.p.c. placental samples (Figure 5C); these data are in contrast to those obtained by Sato *et al.*[12]. and Lin *et al.*[22], who found similar moderate levels of DNA methylation on both the maternal and paternal *Gtl2*-DMR in 6.5, 7.5, and 16.5 d.p.c. extraembryonic tissue.

### CpG dyads within the *Dlk1*-DMR display a high level of hemimethylation

The use of a hairpin linker to covalently attach the complementary strands of DNA prior to bisulfite mutagenesis allowed us to examine the DNA methylation status of complementary sites at CpG dinucleotides. We analyzed a total of 5,965 CpG dyads in embryonic, neonatal, and adult DNA. A total of 1,953 (32.7%) of the CpG dyads analyzed contained methylated cytosines. We observed homomethylation at 1,272 sites (65.1%) and hemimethylation at 681 sites (34.9%). While many of the hemimethylated sites were found on sparsely methylated subclones, hemimethylation was also observed at a higher than expected frequency on densely methylated subclones. For example, among the 55 independent subclones we derived from BxC and CxB adult liver DNA, 21 subclones were methylated at 65% to 100% of the CpG dinucleotides. The total number of CpG dyads with DNA methylation in these densely methylated subclones was 269, 87.4% of which were homomethylated (235) and 12.6% of which were hemimethylated (34) (Table 2).

**Table 2:** **Extent of homo- *vs. *** **hemimethylation at CpG dyads in densely methylated subclones**

	**BxC9C12 adult liver**		**C9C12xB adult liver**	
	**Paternal**	**Maternal**	**Paternal**	**Maternal**
Independent subclones analyzed (*n*)	10	9	15	21
Subclones with >65% methylation (*n*)	8	5	8	0
Methylated dyads (*n*)	95	65	109	N/A
Homomethylated dyads (*n*,%)	89 (93.7%)	54 (83.1%)	92 (84.4%)	N/A
Hemimethylated dyads (*n*,%)	6 (6.3%)	11 (16.9%)	17 (15.6%)	N/A

## Discussion

Proper regulation of imprinted genes is required for normal growth and development in mammals. Loss of imprinting has been shown to result in developmental disorders and disease such as Beckwith-Wiedemann syndrome, which is associated with fetal growth defects, and Prader-Willi and Angelman syndromes, both of which affect neurological development [23]. The regulation of imprinted gene expression is complex and involves various factors, including epigenetic modifications, such as DNA methylation and histone modifications, as well as the activity of long non-coding RNAs and trans-acting factors such as CTCF [3]. The *Dlk1-Dio3* imprinting cluster does not contain CTCF binding sites, and while it does include a maternally expressed long non-coding RNA, *Gtl2*, it is unlikely that *Gtl2* expression regulates the paternally expressed *Dlk1*, as there is limited overlap in the expression patterns of these genes [24,25]. In contrast, differentially methylated regions have been shown to play an important role in the regulation of imprinted expression within the *Dlk1*-*Dio3* cluster, highlighting the critical role epigenetic modifications play in the regulation of genomic imprinting. For example, deletion of the imprinting control region, IG-DMR, from the maternal chromosome results in its paternalization [19].

In addition to regulating the expression of imprinted genes in the *Dlk1-Dio3* cluster, the IG-DMR also influences the acquisition of paternal allele-specific DNA methylation at the secondary *Gtl2*-DMR. It has been shown that the methylation status of the *Gtl2*/*MEG3*-DMR is dependent on the methylation status at the IG-DMR, and that inappropriate hypermethylation of the *Gtl2*/*MEG3*-DMR is concordant with loss of expression [10,20,21]. These data point to a direct role for secondary DMRs in the regulation of imprinted gene expression, although the observation that secondary DMRs acquire differential methylation after the onset of imprinted expression has led to the hypothesis that secondary DMRs play a role in the maintenance of imprinted expression rather than its establishment [6-8]. To date, this study is the first to examine the temporal acquisition of DNA methylation at multiple secondary DMRs within the same imprinting cluster. Our data illustrate that the timing of post-fertilization DNA methylation acquisition is coordinated across the *Dlk1-Dio3* locus, although methylation at the *Dlk1* locus appears more labile (data herein) [11].

Paternal allele-specific methylation at the *Dlk1*-DMR is more variable than at many other imprinted loci, in that the total level of methylation on an individual paternally-inherited allele ranges from 0% to close to 100% at essentially all developmental stages analyzed. Some of this variation may be attributed to the pattern of DNA methylation acquisition at this locus, which appears to be dynamic throughout development. It is also possible that tissue-specific differences result in the variable DNA methylation patterns we observed in whole embryos. For example, *Dlk1* is expressed at high levels in skeletal muscle, a tissue in which imprinting is relaxed, which could correlate with reduced levels of DNA methylation [12,25]. However, even in tissues that display high levels of total DNA methylation on some paternal alleles, such as adult liver, other paternal alleles show little to no methylation and the reason for these differences is not clear. Furthermore, although there are some correlations, there does not appear to be a direct relationship between the DNA methylation profile at the *Dlk1*-DMR and *Dlk1* expression. In most tissues, *Dlk1* expression is restricted to the paternal allele, although there is a relaxation of imprinting in 6.5 d.p.c. embryos and in skeletal muscle, in which 20% and 17% of the expression is derived from the maternal allele, respectively [5,12,15,25]. *Dlk1* is expressed at relatively low levels in early embryos, as compared to the high levels of expression detected in various mid- and late-gestation embryonic tissues such as the pituitary gland, skeletal muscle, liver, and lung [12,25,26]. Despite these differences in expression, our analyses illustrated that the median levels of DNA methylation on the paternal allele is not significantly different in 6.5 to 14.5 d.p.c. whole embryos (Figures 2, 3; Table 1). Finally, while *Dlk1* expression is downregulated in most tissues during late embryogenesis, there was no direct correlation between DNA methylation and *Dlk1* expression levels in tissues derived from 18.5 d.p.c. uniparental disomies [5], nor did we detect a direct correlation in this study. Together, these data suggest that the DNA methylation status at the *Dlk1*-DMR, located in exon 5, may not play an important role in the regulation of expression at this locus. In contrast, the methylation status of the *Gtl2/MEG3*-DMR has been shown to directly influence expression of *Gtl2* in cis, consistent with its location at the *Gtl2* promoter [5,10,20,21]. The critical regulatory role of the *Gtl2*-DMR may explain the maintenance of high average DNA methylation levels at this locus once it has been established [5,11,12]. It is possible that DNA methylation at the *Dlk1*-DMR may reflect a broader, locus-wide epigenetic profile that encompasses both *Gtl2* and *Dlk1*.

### The *Dlk1*-DMR displays low methylation fidelity

The approach we utilized allowed us to analyze the methylation pattern for complementary CpG dinucleotides within the *Dlk1*-DMR. To the best of our knowledge, this is the first study to comprehensively examine the methylation status of complementary CpG dinucleotides at an imprinted gene during development. Of the 1,953 methylated CpG dyads, 1,272 (65.1%) were homomethylated, while 681 (34.9%) were hemimethylated. This result was unexpected, as the fidelity with which the maintenance DNA methyltransferase in mouse, Dnmt1, has been shown to be greater than 95% [27,28]. There are several possible reasons to explain some of the hemimethylation we detected. It is likely that some of the hemimethylated sites we observed are a result of hybrid subclones, which have been shown to result as an artifact of PCR amplification following bisulfite mutagenesis [29]. It is also possible that some of the observed hemimethylation is a result of *Taq*-induced PCR error during amplification. However, these artifacts are unlikely to account for the high level of hemimethylation we detected. Rather, the high level of hemimethylation we observed challenges the idea that Dnmt1 functions with high fidelity at all genomic locations.

A large-scale study analyzing the *in vivo* regulation of CpG methylation by DNA methyltransferases was recently conducted by Arand *et al.*[30]. In this study, the authors found relatively high levels of hemimethylated CpGs in embryonic liver, ranging from 16.2% to 30.6% of the methylated CpG dyads. Interestingly, this work illustrated the relative stability of homomethylation at the imprinted *Snprn* and *H19* genes, but demonstrated high levels of hemimethylation at the imprinted *Igf2* gene (22%). Analyses of DNA methylation profiles in *Dnmt*-mutant embryonic stem cells indicated that the DNA methylation profiles at *Snprn* and *H19* were dependent on the activity of Dnmt1 alone, while maintenance of DNA methylation at *Igf2* required the coordinated activity of Dnmt1, Dnmt3a, and Dnmt3b, a possible consequence of 5-hydroxymethylcytosine enrichment at the *Igf2* DMR [30]. It is therefore possible that the high level of hemimethylation we observed at the *Dlk1*-DMR may be due to the presence of 5-hydroxymethylcytosine at this locus, preventing high levels of fidelity via Dnmt1. An analysis of methylcytosine *versus* 5-hydroxymethylcytosine levels at the *Dlk1*-DMR will address this possibility.

An alternative hypothesis to explain the high level of hemimethylation we observed at the *Dlk1*-DMR is that there may be a lower level of fidelity associated with the maintenance of DNA methylation at secondary DMRs. Consistent with this hypothesis, a study by Vu *et al.*[31] examined DNA methylation on the top and bottom strands of the human *Igf2/H19* imprinted region. Vu and colleagues analyzed DNA methylation on the top and bottom strands separately and found uniform levels of methylation present at the primary DMR. In contrast, they observed less uniformity in the methylation of the top and bottom strands at the *H19* promoter, which is categorized as a secondary DMR as it loses and then regains paternal allele-specific methylation during pre- and post-implantation development, respectively [32,33]. Additionally, a more recent survey of differentially methylated regions associated with imprinted genes in humans support this hypothesis. Woodfine *et al.*[34] reported a higher level of stability for DNA methylation at gametic DMRs than at secondary DMRs. Further examination of CpG dyad methylation patterns at imprinted loci may provide additional insight into the mechanisms responsible for the acquisition and maintenance of DNA methylation at these sites.

## Conclusions

Our analysis of DNA methylation at the mouse *Dlk1*-DMR illustrates that the acquisition of paternal allele-specific DNA methylation initiates between 3.5 and 6.5 d.p.c., suggesting that epigenetic modifications across the *Dlk1-Dio3* imprinting cluster may be coordinately regulated during post-implantation development. The range of DNA methylation levels on individual alleles at the same developmental stage as well as the additional acquisition of DNA methylation on the maternal *Dlk1* allele in adult tissues suggest that the DNA methylation profile of this secondary DMR is more variable than is commonly seen at imprinted loci. We further observed a high level of hemimethylation at the *Dlk1*-DMR: 35% of CpG dyads containing methylated residues were methylated on only one of the two complementary strands. This result is significant because it challenges the idea that Dnmt1 functions with high fidelity at all genomic locations. We hypothesize that the low DNA methylation fidelity we observed is related to the variable DNA methylation profiles at the *Dlk1*-DMR, and may be a consequence of high levels of 5-hydroxymethylcytosine at this locus. These data provide insight into a novel epigenetic profile that may distinguish primary DMRs from secondary DMRs.

## Methods

### Mice

C57BL/6 J (B6) and *Mus musculus castaneus* (CAST) mice were purchased from the Jackson Laboratory. To facilitate the isolation of F_1_ hybrid mice, a strain of mice that served as the source of the *M. m. castaneus* allele (CAST12) was constructed as previously described [11]. Natural matings between B6 and CAST were used to generate F_1_ hybrid males for spermatozoa collection; all other F_1_ hybrid tissues used for bisulfite analyses were generated from natural matings between B6 and CAST12 mice. For all F_1_ hybrid tissues, the maternal allele is located on the left. Ethical approval for procedures involving animals was granted by the Bryn Mawr College Institutional Animal Care and Use Committee, PHS Welfare Assurance Number A3920-01.

### DNA purification and bisulfite analysis

For bisulfite analysis of 3.5 and 6.5 d.p.c. DNA, two to four embryos were pooled prior to digestion with proteinase K. The resulting DNA was subjected to bisulfite mutagenesis using an EZ DNA methylation-direct kit (Zymo Research, cat# D5020). For all other tissues, genomic DNA extractions were performed either from a pool (four 7.5 d.p.c. embryos) or from single embryos, fetuses, or tissues according to the DNeasy protocol (Qiagen) or using a series of phenol/chloroform extractions as described previously [33], and the complementary strands were covalently attached prior to bisulfite mutagenesis as follows: 0.5 μg of genomic DNA was digested with 1 μL *Bgl*I (NEB, cat# R0143S) and ligated to 1 μg of a phosphorylated hairpin linker (5′-AGCGATGCGTTCGAGCATCGCTCCC-3′) [35]. A total of 0.5 μg of hairpin linked-ligated DNA was denatured by incubating in freshly prepared 3 M NaOH for 20 min at 42°C, then subjected to bisulfite mutagenesis using an EZ DNA methylation-direct kit, as above. All mutagenized DNAs were subjected to multiple independent PCR amplifications to ensure analysis of different strands of DNA; subclones derived from independent PCR amplifications are distinguished by different letters of the alphabet. Data from multiple individuals at the same developmental stage were combined, as we did not detect variation between biological replicates. The following primer pairs were used for nested amplification of the mutagenized DNA, and were designed to incorporate both the SNP and at least 50% of the CpG dinucleotides within the CpG island. All base pair numbers are from GenBank Accession Number NC_000078.6. For the first round of amplification of mutagenized 3.5 and 6.5 d.p.c. DNA, two cycles of 94°C for 2 min, 52°C for 1 min, 72°C for 1 min followed by 30 cycles of 94°C for 30 s, 52°C for 1 min, 72°C for 1 min using primers RDlke5BF3 (5′-CCCCATCTAACTAATAACTTACA-3′)/RDlke5BR3 (5′-GTGTTTAGTATTATTAGGTTGGTG-3′). For the second round of amplification, 35 cycles of 94°C for 30 s, 52°C for 1 min, 72°C for 1 min using primers RDlke5BF4 (5′-ATTTCTACTACTCTATCCTAACCC-3′)/RDlke5BR4 (5′-TTAGGATGGTGAAGTAGATGGT-3′) yielded a 597 bp product. To amplify mutagenized DNA treated with the hairpin linker, the same reaction conditions were used with the following primers to yield a 464 bp product: first round, RDlke5BR4 (5′-TTAGGATGGTGAAGTAGATGGT-3′)/Dlk1e5BR1 (5′-AACTCTTTCATAAACACCTTCAA-3′); second round, HPDlk1e5F (5′-GTTTATTTGGGTGTGTTGGAGG-3′)/HPDlk1e5R (5′-AAACTCACCTAAATATACTAAAAAC-3′). The following primer pairs were used for nested or semi-nested amplification of IG- and *Gtl2*-DMRs, as previously described [11]. All base pair numbers are from NC_000078.5. *Gtl2* IG-DMR, with the first nucleotide of IG-BS-F1 corresponding to position 110,766,235: 30 cycles of 94°C for 30 s, 52°C for 1 min, 72°C for 1 min, using primers IG-BS-F1/IG-BS-R, followed by 35 cycles using IG-BS-F2/IG-BS-R and the same cycling conditions as above. Identical reaction conditions were used to amplify the *Gtl2-*DMR, with the first nucleotide of Gtl2BI4F1 corresponding to position 110,779,293: Gtl2BI4F1/Gtl2BI4R1 followed by Gtl2BI4F2/Gtl2BI4R2. Primer sequences follow. IG-BS-F1, 5′-GTATGTGTATAGAGATATGTTTATATGGTA-3′; IG-BS-F2, 5′-GTGTTAAGGTATATTATGTTAGTGTTAGGA-3′; IG-BS-R, 5′-GCTCCATTAACAAAATAATACAACCCTTCC-3′; Gtl2BI4F1, 5′-GAAGAATTTTTTATTTGGTGAGTGG-3′; Gtl2BI4F2, 5′-GTTTGAAAGGATGTGTAAAAATG-3′; Gtl2BI4R1, 5′-CAACACTCAAATCACCCCCC-3′; Gtl2BI4R2, 5′-GCCCCCCACATCTATTCTACC-3′. Subcloning of amplified products was achieved using a pGEM-T Easy vector (Promega Corporation, Madison, WI, USA). Sequencing reactions were performed using a Thermo Sequenase Cycle Sequencing Kit (USB Corporation, Cleveland, OH, USA), and reactions were analyzed on a 4300 DNA Analyzer (LI-COR Biosciences, Lincoln, NE, USA). Percent methylation was calculated based on data obtained from both complementary strands.

### Identification of CpG island

The extent of the CpG island identified by Paulsen *et al.*[24] was determined using the EMBOSS CpGPlot analyzer (http://www.ebi.ac.uk/Tools/emboss/cpgplot/index.html), with the following parameters: program = cpgplot, window = 200, step = 1, obs/exp = 0.6, MinPC = 50, length = 200. The position of the CpG island corresponds to nucleotides 109,459,650-109,460,035 (GenBank: NC_000078.6).

## Abbreviations

B6: C57BL/6; C or CAST: *Mus musculus castaneus*; C12 or CAST12: *Mus musculus castaneus* chromosome 12 on a C57BL/6 background; DMR: Differentially methylated region; d.p.c.: Days post coitum; d.p.p.: Days post partum; ICR: Imprinting control region; PCR: Polymerase chain reaction; UPD: Uniparental disomy.

## Competing interests

The authors declare that they have no competing interests.

## Authors’ contributions

MQ participated in experimental design and carried out molecular genetic studies. AG, AH, CT, JA, and KM carried out molecular genetic studies. TLD conceived of the study and experimental design, carried out molecular genetic studies, and wrote the manuscript. All authors read and approved the final manuscript.

## Supplementary Material

Additional file 1 **Data used for statistical analyses of DNA methylation levels at the *Dlk1*-DMR during different stages of mouse development.** This file contains the numerical data used to perform Kruskal-Wallis and Mann–Whitney U tests. Data from each of the developmental stages are presented in chronological order, as they are in the Results, Figures, and Table 1. Each dataset presents the information for a specific tissue, cross (maternal allele x paternal allele), and parental allele analyzed, as indicated in columns B-D.% methylation (column E) was calculated by dividing the number of methylated CpG sites observed in a given subclone (column A) by the total number of CpG sites analyzed within the subclone; the raw data used to make these calculations are found in Figures 2, 3, 4, and 5. Click here for file

Additional file 2 **Average levels of DNA methylation on the paternal and maternal *Dlk1*-DMR alleles during development, including median values and IQ ranges.** This file expands on the information presented in Table 1. In addition to presenting the average levels of DNA methylation at each developmental stage, Additional file 2 contains median values and IQ ranges. Data from each developmental stage are presented in chronological order, as they are in the Results and Figures. Click here for file
