# Supplementary material for: Analysis of DNA methylation acquisition at the imprinted Dlk1 locus reveals asymmetry at CpG dyads
Source: Epigenetics Chromatin. 2014 May 29;7:9. doi: 10.1186/1756-8935-7-9 (PMC4045959; doi:10.1186/1756-8935-7-9)
Supplement: Additional file 2 — Average levels of DNA methylation on the paternal and maternal Dlk1 -DMR alleles during development, including median values and IQ ranges. This file expands on the information presented in Table 1. In addition to presenting the average levels of DNA methylation at each developmental stage, Additional file 2 contains median values and IQ ranges. Data from each developmental stage are presented in chronological order, as they are in the Results and Figures. [file 1756-8935-7-9-S2.docx]

**Additional File 2.** Average levels of DNA methylation on the paternal and maternal *Dlk1*-DMR alleles during development, including median and IQ ranges.

| genomic DNA sample | % methylation,  paternal alleles* | median | IQ range | % methylation,  maternal alleles* | median | IQ range |
| --- | --- | --- | --- | --- | --- | --- |
| B6xCAST adult sperm | 12% | 0.074 | 0.016-0.157 | 9% | 0.094 | 0.000-0.156 |
| 3.5 d.p.c. B6xCAST12 embryo | 4% | 0.015 | 0.000-0.029 | 0.5% | 0.000 | 0.000-0.000 |
| 6.5 d.p.c. B6xCAST12 embryo | 36% | 0.372 | 0.243-0.464 | 14% | 0.194 | 0.063-0.194 |
| 7.5 d.p.c. B6xCAST12 embryo | 35% | 0.376 | 0.219-0.532 | 10% | 0.172 | 0.000-0.250 |
| 8.5 d.p.c. B6xCAST12 embryo | 22% | 0.188 | 0.059-0.288 | 5% | 0.032 | 0.000-0.096 |
| 9.5 d.p.c. B6xCAST12 embryo | 37% | 0.251 | 0.112-0.592 | 10% | 0.031 | 0.000-0.187 |
| 14.5 d.p.c. B6xCAST12 embryo | 27% | 0.156 | 0.064-0.477 | 5% | 0.031 | 0.000-0.032 |
| 14.5 d.p.c. CAST12xB6 embryo | 21% | 0.219 | 0.079-0.297 | 3% | 0.031 | 0.000-0.036 |
| 17.5 d.p.c. CAST12xB6 liver | 74% | 0.875 | 0.604-0.938 | 18% | 0.219 | 0.000-0.403 |
| 5 d.p.p. B6xCAST12 liver | 46% | 0.467 | 0.305-0.531 | 11% | 0.016 | 0.000-0.041 |
| 5 d.p.p. CAST12xB6 liver | 62% | 0.813 | 0.344-0.891 | 24% | 0.180 | 0.000-0.516 |
| 6 d.p.p. B6xCAST12 lung | 45% | 0.356 | 0.225-0.649 | 8% | 0.033 | 0.000-0.067 |
| 5 d.p.p. CAST12xB6 lung | 44% | 0.409 | 0.376-0.485 | 14% | 0.032 | 0.000-0.126 |
| adult B6xCAST12 liver | 74% | 0.739 | 0.659-0.811 | 57% | 0.688 | 0.375-0.742 |
| adult CAST12xB6 liver | 58% | 0.688 | 0.516-0.813 | 41% | 0.500 | 0.344-0.563 |
| adult B6xCAST12 lung | 18% | 0.125 | 0.094-0.167 | 6% | 0.052 | 0.000-0.105 |
| adult CAST12xB6 lung | 53% | 0.250 | 0.094-0.563 | 32% | 0.500 | 0.407-0.766 |

*A Kruskal-Wallis test was used to assess overall variation among paternal alleles and among maternal alleles. Significant variation was observed among both the paternal alleles (P=8.335e-13) and the maternal alleles (P=1.844e-12).
